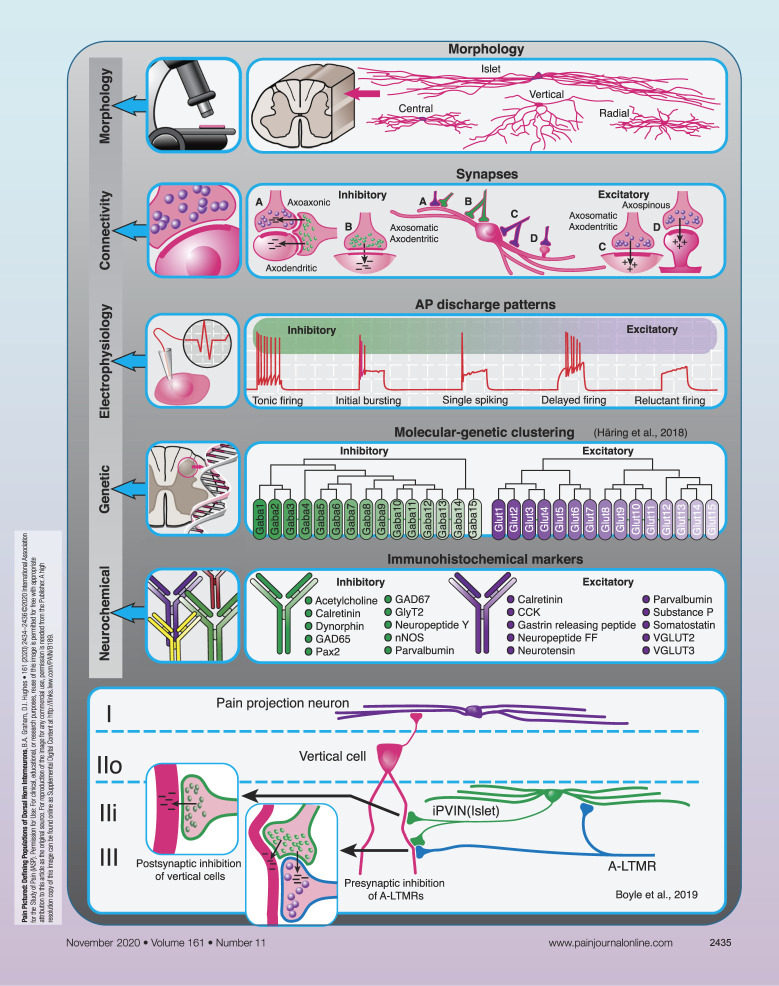# Defining populations of dorsal horn interneurons

**DOI:** 10.1097/j.pain.0000000000002067

**Published:** 2020-10-13

**Authors:** Brett A. Graham, David I. Hughes

**Affiliations:** a School of Biomedical Sciences and Pharmacy, University of Newcastle, Newcastle, NSW, Australia; b Spinal Cord Research Group, Institute of Neuroscience and Psychology, University of Glasgow, Glasgow, United Kingdom

Neuronal circuits within the spinal dorsal horn play a crucial role in how we ultimately perceive sensory inputs, and disruption to these circuits can lead to altered sensation and chronic pain states. To understand how these circuits process sensory information in health and disease, it is necessary to identify the individual neuronal components, determine how they are connected, and define the functional significance of these pathways. Here, we provide an overview of current strategies used to better define interneuron populations and the circuits they form within the dorsal horn.

Interneurons account for 99% of all neurons in the spinal dorsal horn^1^ and play critical roles in modulating modality-specific circuits to ultimately affect our perception of afferent input.^22^ These cells represent a potential target for pharmacological intervention to manage various forms of chronic pain, but to achieve this we must first define their contributions to distinct spinal circuits under normal conditions and determine how these change in pathological states. Given that dorsal horn interneurons are diverse in terms of their morphological, neurochemical, and functional properties, identifying a series of distinguishing features to characterise discrete populations can be challenging. In this PAIN Pictured article, we outline key features that can be used to define distinct interneuron groups and provide an example where this approach has helped identify a population of spinal interneurons that are implicated in the development of tactile allodynia.

Spinal interneurons can be subdivided into 2 principal classes based on their neurotransmitter content: excitatory interneurons release glutamate, whereas inhibitory interneurons use GABA and/or glycine. In both the rat and mouse, inhibitory interneurons account for approximately 25% of all neurons in laminae I and II and 40% of those in lamina III.^14,15,19^ Both interneuron classes can be further subdivided into distinct subpopulations based on their morphological, electrophysiological, neurochemical, and genetic characteristics.^2,3,7–9,11,17,18,23,24^ For example, in lamina II of the rodent spinal cord, 4 morphologically distinct populations of neurons (islet, radial, central, and vertical cells) have been described, although other cells that do not conform to these classes (unclassified neurons) are also relatively common.^7,23,24^ Islet cells are considered to comprise a subpopulation of inhibitory interneurons, whereas the remaining morphologies are common among excitatory interneurons.^11,24^

Like neuronal morphology, synapses formed by excitatory and inhibitory interneurons show distinct ultrastructural features. Axon terminals from glutamatergic interneurons typically contain rounded agranular synaptic vesicles and form asymmetrical synapses onto cell bodies (axosomatic synapses), dendrites (axodendritic synapses), or dendritic spines (axospinous synapses). Axon terminals from inhibitory interneurons contain flattened (pleomorphic) synaptic vesicles and form symmetrical synapses onto cell bodies and dendrites primarily but can also form axoaxonic synapses onto the central terminals of most classes of sensory afferents (with the exception of peptidergic C fibres).^2,16^ Axoaxonic synapses are the anatomical basis of presynaptic inhibition and exert strict control on the passage of sensory information relayed at the first central synapse. Axon terminals from inhibitory interneurons often form synaptic triads by establishing axoaxonic synapses onto the central terminals of sensory fibres and also axodendritic synapses onto dendrites that are themselves postsynaptic to the same primary afferent terminal, providing a powerful mechanism to regulate afferent input in the dorsal horn.^16^ Anatomical features can therefore provide valuable insight into the role of individual cells in spinal circuits, but identifying the neuronal population that give rise to the various types of synapses in the dorsal horn remains challenging.

Electrophysiological properties are also used to distinguish dorsal horn neurons. For example, 5 distinct action potential discharge patterns in response to current injection are routinely described in lamina II neurons (tonic, delayed, and reluctant firing, as well as initial bursting and single spiking).^7,17^ These discharge patterns often correlate with cell morphology, where islet cells typically exhibit tonic firing or initial bursting, and vertical cells show delayed-firing patterns.^7,23,24^ As with morphology, however, action potential discharge classification requires caution as some responses fall outside the patterns commonly described.^17^ These response patterns can also vary depending on membrane potential,^23^ the laminae in which responses have been recorded,^2^ and whether recordings were performed in vitro or in vivo.^6^

Attempts to identify distinct populations of dorsal horn interneurons have assessed correlations between morphological and electrophysiological properties,^11,23,24^ whereas other approaches use neurochemical expression patterns to help define cells within this region. For example, various neuropeptides, calcium-binding proteins, enzymes, and amino acid transporters have helped define both excitatory and inhibitory interneuron subpopulations in the dorsal horn based on the neurochemical content.^3,8,20^ More recently, molecular and genetic profiling has broadened the spectrum of molecular markers that can be used to define distinct neuronal populations.^2,9,18^ When coupled with the increasing availability of different transgenic mouse lines, viruses, and experimental approaches to label, manipulate, and control these cells, researchers are now well placed to define spinal circuits responsible for acute and chronic pain.

Current models of how interneuron populations modulate spinal sensory processing have their origins in Melzack and Wall's gate control theory of pain, where a spinal circuit that influences the relaying of pain signals to higher brain centres is described.^12^ In this circuit, inhibitory interneurons act directly on the central terminals of both large- and small-diameter primary afferents, through axoaxonic inputs, to modulate input directly. Given that axoaxonic synapses have been established on most classes of afferent fibres,^1,2,16,20^ surprisingly little progress has been made in identifying the cells that give rise to these connections. We have recently combined the above approaches to identify parvalbumin-expressing inhibitory interneurons (iPVINs) as a source of axoaxonic input onto myelinated low-threshold mechanoreceptor afferents (ALTMRs). These iPVINs mediate both presynaptic inhibition of ALTMR terminals and postsynaptic inhibition of several populations of cells targeted by ALTMRs, including vertical cells.^4,10^ Vertical cells comprise a population of excitatory interneurons that relay information to spinoparabrachial projection neurons in lamina I^5^ but have also been proposed as a route through which ALTMR input can activate pain circuits under pathological conditions.^21,25^ Targeted manipulation of PVINs using chemogenetic, optogenetic, and cell-ablation approaches has established the functional significance of these cells^13^ and shows that a reduction in the inhibition they mediate results in the development of mechanical hypersensitivity. The precise mechanism underlying spinal disinhibition after peripheral nerve injury remains the subject of debate,^4,13,20,21,25^ but recent evidence implicates changes in the intrinsic excitability of iPVINs rather than a loss of their synaptic connections.^4^ Nonetheless, a reduction in spinal inhibition can unmask otherwise dormant circuits, and these can lead to aberrant activation of pain circuits.^4,21,25^ Together, these multidisciplinary studies identify iPVINs as critical components in microcircuits that gate ALTMR input under normal conditions^4,10,13^ and serve to present these cells as potential therapeutic targets to alleviate tactile allodynia.

## Conflict of interest statement

The authors declare no conflict of interest but acknowledge funding provided by the NHMRC, Australia (grants 631000, 1043933, 1144638, and 1184974 to B.A.G), the Hunter Medical Research Institute (B.A.G.), and the BBSRC (grants BB/J000620/1 and BB/P007996/1 to D.I.H.).

## Figures and Tables

**Figure:**